# The m^6^A demethylase FTO targets POLQ to promote ccRCC cell proliferation and genome stability maintenance

**DOI:** 10.1007/s00432-023-05541-0

**Published:** 2024-01-25

**Authors:** Yichen He, Yimeng Chen, Zhengsheng Li, Changping Wu

**Affiliations:** 1 Department of Tumor Biological Treatment, The Third Affiliated Hospital of Soochow University, Changzhou, 213003 China; 2 Institute of Cell Therapy, The Third Affiliated Hospital of Soochow University, Changzhou, 213003 China; 3 Department of Urology, The Third Affiliated Hospital of Soochow University, Changzhou, 213003 China; 4 Department of Oncology, The Third Affiliated Hospital of Soochow University, Changzhou, 213003 China

**Keywords:** m^6^A, ccRCC, FTO

## Abstract

**Background and aim:**

As the first identified m^6^A demethylase, FTO has been implicated in the progression of various cancers. However, the specific mechanism of FTO in clear cell renal cell carcinoma (ccRCC) remains incompletely understood. In this study, we aimed to explore the potential molecular mechanisms influencing the progression of ccRCC.

**Methods:**

We initially assessed the expression of FTO in tumor and adjacent tissues using TCGA database, RT-qPCR, and Western blot. We then conducted CCK-8, cell cycle analysis, and colony formation assay to investigate the impact of FTO on ccRCC cell proliferation. MeRIP-seq and RNA-seq were employed to identify potential downstream targets of FTO in ccRCC, and these findings were further validated through dual-luciferase reporter assays and MeRIP-qPCR. Then, DNA damage and cell death were assessed separately through gammaH2AX immunofluorescence detection and the LIVE/DEAD Fixable Dead Cell Stain assay, respectively. Subsequently, we identified downstream pathways influenced by FTO's regulation of POLQ through TCGA database analysis and GSEA enrichment analysis. Validation was carried out through Western blot.

**Results:**

FTO is highly expressed in ccRCC tissues and cell lines. Furthermore, ROC curve demonstrates that FTO contributes to the diagnosis of ccRCC. FTO modulates m^6^A modification, consequently influencing the expression of POLQ, thus facilitating cell proliferation and maintaining genome stability in ccRCC.

**Conclusion:**

FTO could potentially serve as a diagnostic marker for ccRCC. FTO promotes the progression of ccRCC by regulating m^6^A modification, making the inhibition of FTO a potential novel therapeutic strategy in ccRCC.

**Supplementary Information:**

The online version contains supplementary material available at 10.1007/s00432-023-05541-0.

## Introduction

Renal cell carcinoma (RCC) is one of the most common tumors in the urinary system, and its incidence has been increasing in recent years. As the most prevalent subtype, clear cell renal cell carcinoma (ccRCC) accounts for approximately 75%–80% of RCC cases (Inamura 2017). Surgical resection and radiofrequency ablation (RFA) are commonly used as treatment for early-stage ccRCC, but still, a quarter of patients experience recurrence after surgery, leading to poor prognosis (Choueiri and Motzer 2017). Tyrosine kinase inhibitors (TKI) and immune checkpoint inhibitors (ICI) are first-line adjuvant drugs for ccRCC treatment, yet due to variations in patient drug resistance, some patients still struggle to benefit from these treatments. Consequently, further elucidation of the molecular mechanisms underlying the malignant progression of ccRCC is still necessary, and the development of relevant targeted therapies is awaited to promote personalized and precision treatment for ccRCC patients.

N6-methyladenosine (m^6^A) modification refers to the methylation of adenosine at the N6 position of RNA. In the majority of eukaryotic cells, m^6^A modification is the most common post-transcriptional modification (Yue et al. 2015).Although m^6^A modification was identified on RNA by researchers in the last century, its biological functions were not widely studied. It was not until 2011 that JIA et al. first discovered that fat and obesity-related gene (FTO) proteins could act as “erasers” of m^6^A modification, making m^6^A modification reversible and dynamically regulated. With the development of high-throughput sequencing and immunoprecipitation techniques, researchers found that m^6^A modification sites possess highly conserved motifs, typically known as the RRACH motif (R: purine, A: adenine, C: cytosine, H: adenine, cytosine, or uracil), and these modification sites are often located at the transcription start site (TSS) and the 3'-untranslated region (3'-UTR). m^6^A modification enzymes include the m^6^A “writers” responsible for adding m^6^A modifications to mRNA, the m^6^A “readers” that recognize modifications and play downstream regulatory roles, and the m^6^A “erasers” that remove m^6^A modifications. FTO regulates autophagy, mitochondrial activity, and even radiotherapy and chemotherapy resistance by affecting the m^6^A modification levels of different downstream target genes in cancers, thereby influencing tumor progression and prognosis (Xu et al. 2022; Zhou et al. 2018; Zhuang et al. 2019).

DNA polymerase θ (POLQ) is a key protein in the microhomology-mediated end joining (MMEJ) pathway, containing a conserved superfamily 2 helicase domain at its N-terminus, a DNA polymerase domain at its C-terminus, and a non-conserved central structure domain in between (Schrempf et al. 2021).MMEJ plays a critical role in DNA double-strand break (DSB) repair mechanisms and sometimes competes with homologous recombination (HR), even becoming the only effective DSB repair pathway under certain conditions (Ceccaldi et al. 2015; Truong et al. 2013). POLQ exhibits elevated expression levels in diverse malignant tumor tissues, such as lung adenocarcinoma, gastric cancer, colorectal cancer, breast cancer, cervical cancer, and oral cancer, and its presence has been linked to unfavorable prognostic outcomes in a number of tumors (Allera-Moreau et al. 2012; Ceccaldi et al. 2015; Lemee et al. 2010; Pillaire et al. 2010). Interestingly, according to recent reports, tumors with HR deficiencies exhibit a particular reliance on DNA damage repair mediated by POLQ. Inhibition of POLQ effectively reverses the resistance of tumors with HR deficiencies to Poly (ADP-ribose) polymerase inhibitors (PARPi) (Zatreanu et al. 2021).While previous studies have established a link between m^6^A level and POLQ expression, the specific regulatory mechanisms remain elusive (Dong et al. 2022).

The m^6^A methylation modification has been demonstrated to regulate DNA damage response (DDR), and FTO has also been found to be indispensable for various DDR functions (Liu et al. 2023; Xiang et al. 2017). This study explored the impact of FTO on the progression of ccRCC and identified elevated FTO expression in tumor tissues. Mechanistic investigations revealed that downregulating FTO could inhibit ccRCC cell proliferation and impair its DDR function. Subsequent analyses indicated that the FTO/m^6^A/POLQ axis contributes to this phenomenon. The findings of this study may offer new therapeutic targets for ccRCC and open avenues for potential treatment strategies.

## Materials and methods

### Cell lines and cell culture

Human ccRCC cell lines, including 786-O, ACHN, 769-P, OSRC-2, and CAKI-1, were procured from the National Collection of Authenticated Cell Cultures, a part of the Cell Bank of the Chinese Academy of Sciences. The cell lines 786-O, ACHN, 769-P, and OSRC-2 were cultured in RPMI 1640; while, CAKI-1 cells were cultured in Mc Coy’s 5A medium. Both RPMI 1640 and Mc Coy's 5A were supplemented with 10% fetal bovine serum, 100 µg/mL penicillin, and 100 µg/mL streptomycin. Cells were cultured at 37 °C in a humidified atmosphere containing 5% CO_2_ and periodically screened for mycoplasma contamination using a mycoplasma detection kit.

### ccRCC tumor and adjacent tissues

A total of 72 ccRCC patients who underwent radical nephrectomy at the Third Affiliated Hospital of Soochow University from 2019 to 2022 were included in this study. Tumor tissues and corresponding adjacent normal tissues (located more than 5 cm away from the tumor) were collected from these patients. Clinical data such as age, weight, gender, tumor stage, and pathological grade were recorded. None of the patients had received local or systemic treatments before surgery. Fresh tumor tissues and matched adjacent normal tissues were preserved at -80°C for subsequent experiments including RT-qPCR and Western blot analysis. The remaining specimens were fixed in formalin, embedded in paraffin, and subjected to pathological examination and immunohistochemical (IHC) analysis. Ethical approval for our study protocol was obtained from the Ethics Committee of the Third Affiliated Hospital of Soochow University.

### Transfection and infection

FTO, POLQ, and non-targeting control (NC) siRNAs were purchased from RiboBio (Guangzhou, China), with their corresponding sequences detailed in Additional file 1 (Table S1). Transient transfections were executed using Lipofectamine 3000 (Invitrogen, USA), following the manufacturer's guidelines. To achieve stable FTO knockdown cells, lentiviruses expressing sh-FTO constructs were constructed by Gene Pharma (Suzhou, China). A negative control shRNA (sh-control) was employed for comparison. The efficacy of si-FTO and sh-FTO was assessed through RT-qPCR and Western blot analysis.

### Western blotting

Proteins were extracted using RIPA lysis buffer containing 1% PMSF and 1% cocktail protease inhibitor. The protein concentration was determined using the BCA assay kit (PC0020, Solarbio, China). The protein samples were separated on a 10% SDS-PAGE gel and then transferred onto a PVDF membrane. The membrane was subsequently incubated with primary antibodies, including FTO (1:2000, ab126605, abcam, UK), POLQ (1:1000, SAB1402530, Merk, Germany), CDC6 (1:2000, ab109315, abcam, UK), CDT1 (1:1000, ab202067, abcam, UK), FEN1 (1:2000, ab109132, abcam, UK), NBN (1:2000, ab32074, abcam, UK), and XRCC1 (1:2000, ab134056, abcam, UK). Subsequently, appropriate secondary antibodies (1:10,000, abcam, UK) were applied for 1 h. The protein bands were visualized using a chemiluminescence imaging system (5200, Tanon, China) for image analysis.

### CCK-8 assay

Cells were counted using an automated cell counter (Countess3, Invitrogen, USA) and subsequently passaged into 96-well plates at a density of 1000 cells per well, with 5 replicates per group. After culturing for 24, 48, and 72 h, the culture medium was replaced with 100 μL of medium containing 10% CCK-8 reagent. Following a 2-h incubation at 37 °C, the absorbance was measured at 450 nm to assess cell viability.

### Colony formation assay

Cells were counted using an automated cell counter (Countess3, Invitrogen, USA) and subsequently seeded in 6-well plates at a density of 1000 cells per well, with three replicates per group. After approximately one week of cultivation, When the cell count in the majority of clones exceeded 50 cells, fixation was carried out using a fixative solution (P0098, Beyotime, China), followed by staining with crystal violet (C0121, Beyotime, China).

### Flow cytometry

Cell cycle analysis was performed following the manufacturer's instructions of the Flow Cytometric Cell Cycle Staining Kit (CCS012, MultiScience, China). Approximately 3 × 10^5^1 × 10^6^ cells were collected, washed with PBS, stained with propidium iodide (PI) at room temperature for 30 min, and subsequently analyzed using the flow cytometer analysis. The use of LIVE/DEAD Fixable Dead Cell Stain Kits (L34973, Thermo Fisher Scientific, America) and CytoTell Red 650 proliferation kits (22,255, AAT Bioquest, America) followed the manufacturer's instructions.

### RNA-seq and MeRIP-seq

Total RNA was extracted using TRIzol Reagent (15,596,026, Introvigen, USA) following the manufacturer's instructions. The assessment of the isolated RNA samples' quality and concentration was performed using the NanoDrop™ One (Invitrogen, USA), measuring the A260/230 and A260/280 ratios, respectively. High-throughput m^6^A and mRNA sequencing were performed as previously described (Chen et al. 2020), carried out by OE Biotech. Co., Ltd. (Shanghai, China).

### Dual-luciferase reporter assays

A dual-luciferase reporter gene assay was performed using the Dual-Luciferase Reporter Gene Assay Kit (11402ES60, Yeasen, China) following the manufacturer's instructions. In brief, wild-type (POLQ-WT) and mutant-type (POLQ-MUT, with the A residue at the m^6^A site replaced by C) plasmids were designed and constructed, and then inserted into the pmirGLO vector. Subsequently, POLQ-WT and POLQ-MUT plasmids were co-transfected with control si-NC and si-FTO, and luciferase activity was determined by dividing the firefly luciferase activity (RLU) by the Renilla luciferase activity (RLU) after transfection, thereby determining the activation levels of different genes.

### m^6^A dot blot assays

Total RNA was extracted from ccRCC cells. After measuring the RNA concentration, 2μL(800ng) of RNA was spotted onto a nylon membrane (GE Healthcare, USA). Subsequently, the membrane was crosslinked with UV light for 10 min and then incubated overnight at 4°C with an m^6^A antibody (1:4000, ab232905, abcam, UK). Following incubation with the secondary antibody, observations were made using an imaging system.

### Immunofluorescence staining

Pre-treated cells were washed with phosphate-buffered saline and fixed with fixation solution (P0098, Beyotime, China) for 20 min, followed by permeabilization with permeabilization solution (P0097, Beyotime, China) for 15 min. After blocking with 3% bovine serum albumin (BSA) for 30 min, cells were incubated overnight with gammaH2AX antibody (1:400, 9718, CST, USA), followed by incubation with donkey anti-rabbit fluorescent secondary antibody (1:500, 4412, CST, USA) for 1 h the next day. Nuclei were counterstained with Hoechst dye (1:1000, C1011, Beyotime, China) for 15 min, and fluorescence analysis was performed using a fluorescence microscope (BX63, Olympus, Japan).

### ccRCC tissue microarray and multiplex immunofluorescence staining

The ccRCC tissue microarray was purchased from Shanghai XinChao Biotech Co., Ltd. Specimens for microarray were obtained from a total of 144 cases of ccRCC tumors.The multiplex immunofluorescence staining assay involved baking the tissue microarray at 60°C, deparaffinization, and rehydration of slides. Antigen retrieval was performed using microwave treatment. Subsequently, primary and secondary antibodies were applied, followed by imaging and quantitative fluorescence analysis using imaging software (ZEN 3.3 and HALO 3.5).

### RNA stability

To assess the RNA stability, ccRCC cells were treated with 8μg/ml actinomycin D (AbMole, USA). At 0, 2, 4 and 8h, RNA was isolated using TRIzol Reagent (15,596,026, Introvigen, USA) for reverse transcription. The transcriptional abundance of POLQ mRNA was then quantified using RT-qPCR.

### Statistical analysis

Data from three or more groups are presented as mean ± standard deviation (mean ± SD). All statistical analyses were performed using GraphPad Prism 8.0.2 software. Student’s t-test was employed for comparisons between cancer and adjacent non-cancer samples. One-way analysis of variance (ANOVA) was used to assess differences among three or more groups. A significance level of *p* < 0.05 was considered statistically significant.

## Results

### m^6^A demethylase enzyme FTO exhibits elevated expression in ccRCC tissues and cell lines

**Fig. 1:**
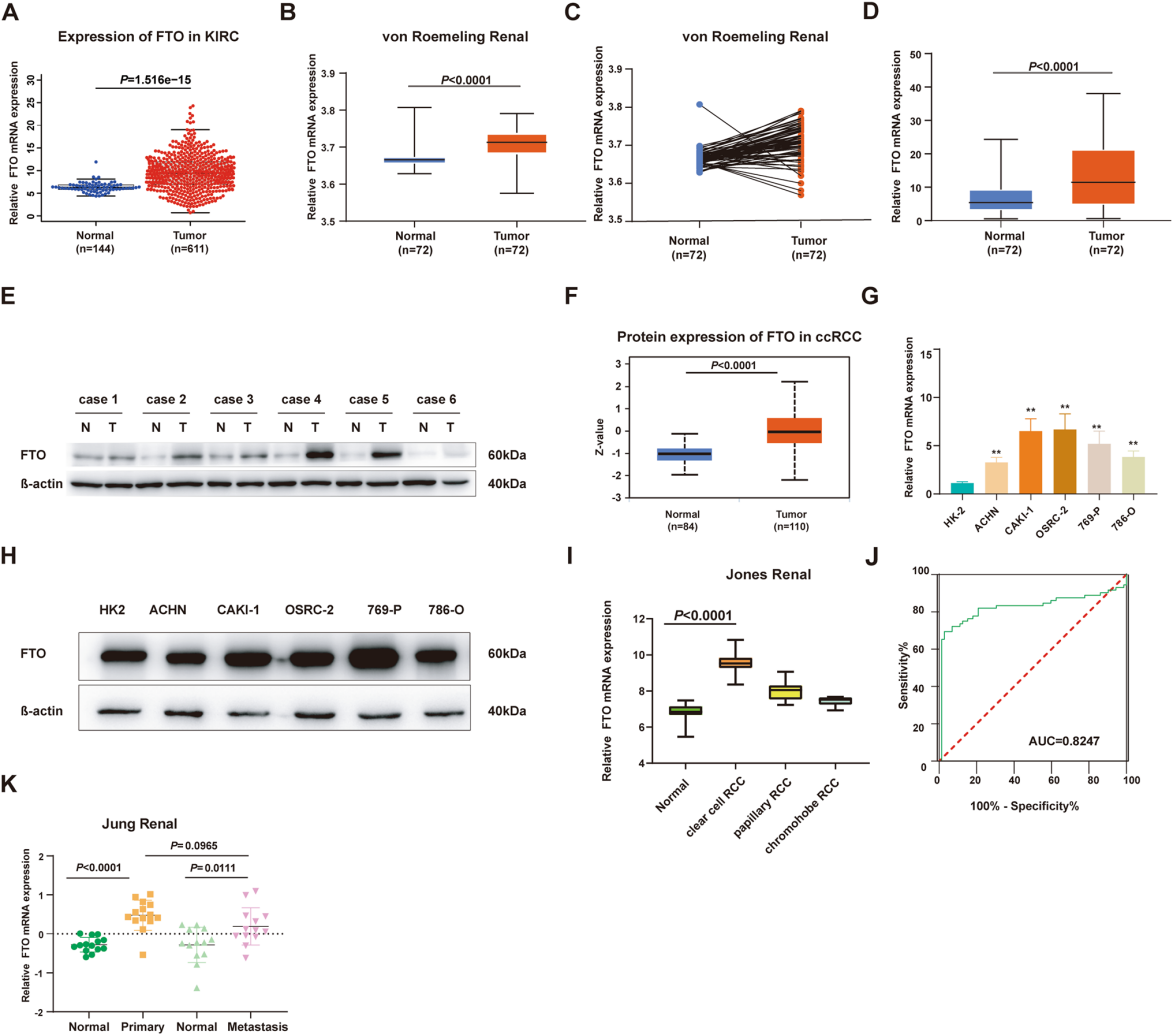
Elevated expression of FTO in ccRCC tissues and cell Lines.** A** FTO mRNA expression in ccRCC and adjacent tissues was examined using the TCGA database. **B, C** FTO mRNA expression in ccRCC and adjacent tissues was assessed through GSE chips (GSE 53757). **D** FTO mRNA expression levels in ccRCC and adjacent tissues were detected in clinical samples. **E** FTO protein expression in ccRCC and adjacent tissues was determined in clinical samples. **F** The CPTAC protein database was used to examine FTO protein expression in ccRCC and adjacent tissues. **G** RT-qPCR was performed to detect FTO mRNA expression in ccRCC cell lines and renal tubular epithelial cell HK2. **H** Western blot was conducted to assess FTO protein expression in ccRCC cell lines and renal tubular epithelial cell HK2. **I** FTO mRNA expression levels in various pathological types of ccRCC were measured using GSE chips (GSE 15641). **J** FTO mRNA expression in ccRCC and adjacent tissues was detected using GSE chips (GSE 53757), and an ROC curve was plotted. **K** FTO expression in primary tumors, cancer tissues, and metastatic cancers of ccRCC was measured using GSE chips (GSE 66270, 66,271). *P < 0.05, **P < 0.01

To investigate the regulatory role of m^6^A-related proteins in ccRCC, we initially collected and examined the expression of key m^6^A modification enzymes in 31 pairs of ccRCC patient tumor tissues and adjacent non-tumor samples by RT-qPCR (Fig. S1). Findings showed that only the mRNA expression of METTL3 and FTO was upregulated in ccRCC tissues. According to the previous reports, ccRCC tissues generally display a decreased overall level of m^6^A modification, making the elevated expression of FTO particularly significant in ccRCC progression (Liu et al. 2022). This high-expression trend of FTO in ccRCC tumor tissues was further confirmed with data from the TCGA database, which included 533 cases of ccRCC tumors and adjacent tissues (Fig. 1A), and high-throughput sequencing chips downloaded from the GEO database (GSE 53757) (Fig. 1B,C). To further verify the expression of FTO in ccRCC, we expanded our clinical sample cohort and performed RT-qPCR, which yielded results consistent with the previous findings (Fig. 1D).

As FTO plays a role in regulating m^6^A modification at the protein level, the protein expression levels of FTO in tumor and adjacent tissues required further investigation, and the results indicated that FTO still exhibited increased protein expression in tumor tissues (Fig. 1E). Furthermore, analysis of the CPTAC protein database revealed high protein expression of FTO in ccRCC tissues (Fig. 1F).

In ccRCC cell lines, FTO mRNA expression levels showed a high degree of consistency with tissue mRNA expression. The mRNA expression of FTO was markedly elevated in all tumor cell lines, with CAKI-1, OSRC-2, and 769-P cells displaying the most significant increases (Fig. 1G). However, Western blot showed that not all ccRCC cell lines displayed an upregulation of FTO protein expression, only CAKI-1 and 769-P cells maintained an upregulation trend consistent with mRNA levels (Fig. 1H).

Additionally, according to the high-throughput sequencing analysis (GSE 15641), among various pathological types of RCC, FTO has the highest mRNA expression level in ccRCC, which may contribute to the differential diagnosis of ccRCC (Fig. 1I). Furthermore, ROC curves generated based on the transcriptome data of ccRCC tumors and adjacent non-tumor tissues demonstrated that FTO also exhibits good diagnostic performance in ccRCC (GSE 53757) (Fig. 1J), suggesting that FTO might be one of the potential driving genes in ccRCC. Further analysis of FTO expression in RCC tissues of different pathological types and stages validated that FTO was specifically upregulated in both primary and metastatic RCC, as evidenced by sequencing data (GSE66270, 66,271). However, FTO expression levels did not distinguish between primary and metastatic tumors (Fig. 1K).

### FTO promotes ccRCC cell proliferation through the regulation of m^6^A modification

**Fig. 2:**
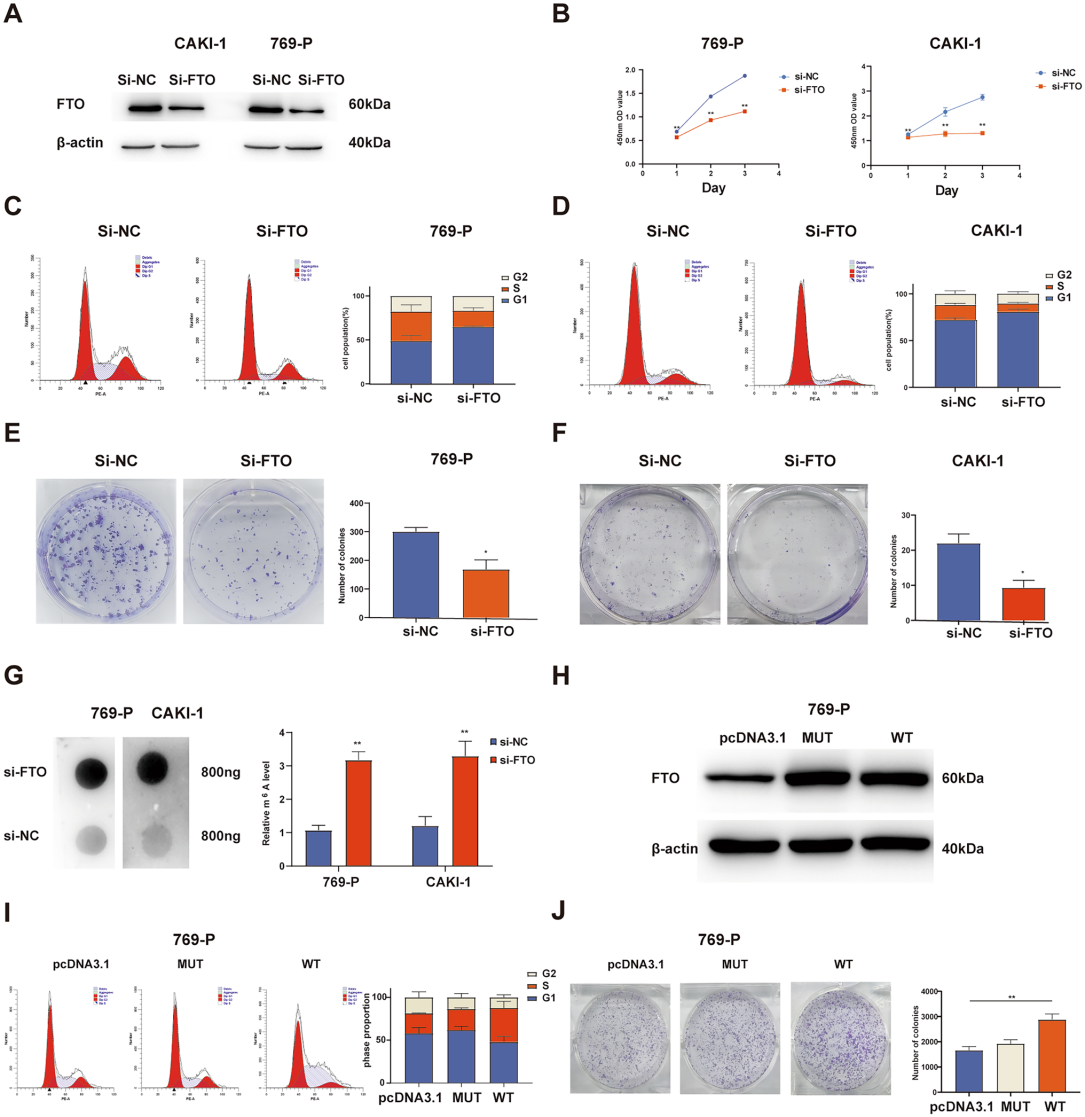
FTO promotes ccRCC cell proliferation by regulating m^6^A modification.** A** Western blot was conducted to assess the transfection efficiency of si-FTO in 769-P and CAKI-1. **B** CCK-8 was performed to evaluate the effect of FTO knockdown on the proliferation of 769-P and CAKI-1. **C, D** Flow cytometry were conducted to examine the influence of FTO knockdown on the cell cycle of 769-P and CAKI-1. **E, F** Colony formation assays was employed to assess the impact of FTO knockdown on the proliferation of 769-P and CAKI-1 cells. **G** m^6^A dot blot analysis was used to determine the m^6^A levels of total RNA. **H** Western blot was performed to evaluate the transfection efficiency of FTO^MUT^ and FTO^WT^.** I** Flow cytometry was conducted to examine the effect of FTO^MUT^ and FTO^WT^ overexpression on the cell cycle of 769-P. **J** Colony formation assays were employed to evaluate the impact of FTO^MUT^ and FTO^WT^ overexpression on the proliferation of 769-P. *P < 0.05, **P < 0.01

Given the consistent expression trend of FTO in the CAKI-1 and 769-P cell lines with that in tumor tissues, these two cell lines were selected for subsequent experiments. FTO was initially knocked down in CAKI-1 and 769-P, and knockdown efficiency was verified through RT-qPCR and Western blot (Fig. 2A). The CCK-8 assay revealed a significant decrease in the 450nm OD values at 24, 48, and 72h after FTO knockdown in both CAKI-1 and 769-P, indicating inhibited cell proliferation (Fig. 2B). Flow cytometry-based cell cycle analysis showed a significant increase in the G1/G0 phase and a decrease in the S phase in CAKI-1 and 769-P after FTO knockdown (Fig. 2C,D). Colony formation assays further confirmed the inhibitory effect of FTO knockdown on ccRCC cell proliferation (Fig. 2E,F). These results collectively demonstrate the inhibitory role of FTO knockdown on ccRCC cell proliferation.

To further investigate whether FTO affects ccRCC tumor cell proliferation through the regulation of m^6^A modifications, we knocked down FTO in CAKI-1 and 769-P and subsequently assessed the overall m^6^A methylation levels in the cells using m^6^A dot blot assay. In both CAKI-1 and 769-P, the overall m^6^A levels significantly increased after FTO knockdown (Fig. 2G). Figure 2H depicts the overexpression efficiency of FTO^WT^ and FTO^MUT^ (with mutations at sites H231A and D233A, disrupting FTO's catalytic functional sites) in 769-P cells (Huang et al. 2022). Cell cycle analysis demonstrated a significant increase in the S phase in the FTO^WT^ group, rather than the FTO^MUT^ group (Fig. 2I). Similar results were observed in colony formation assays, where the FTO^WT^ group exhibited a significantly higher number of colonies compared to the control group (Fig. 2J). Based on these results, it is evident that FTO regulates ccRCC cell proliferation through its catalytic domain involved in m^6^A modifications.

### POLQ serves as a direct downstream target of FTO

To elucidate the downstream targets modulated by FTO in ccRCC, stable low-expressing FTO cell lines (769-P LV-SH FTO) were established by transfecting plasmids carrying FTO shRNA into 769-P cells, followed by a two-week selection period with 1 μg/ml puromycin. The infection efficiency were confirmed through observation using fluorescence microscopy (Fig. S2A). A significant reduction in FTO protein expression was observed in 769-P LV-SH FTO (Fig. S2B). RNA extracted from 769-P LV-SH FTO and 769-P LV-SH control was used for MeRIP-seq and RNA-seq sequencing. RNA-seq revealed consistent gene expression patterns within their respective groups (Fig. S3A). After FTO knockdown, among the genes with differential expression more than 1.5-fold, there were 596 upregulated genes and 467 downregulated genes (Fig. S3B). GO analysis revealed that differentially expressed genes were enriched in biological processes related to cell proliferation, including spindle assembly, sister chromatid separation, and cell division (Fig. S3C, D). KEGG analysis showed that the enriched pathways for differentially expressed genes included cancer-related pathways, including cell proliferation, cell death, DNA replication, and DNA damage repair pathways (Fig. S3E, F).

MeRIP-seq results indicated that m^6^A modification sites were commonly located in exon regions, with the majority situated near the mRNA 3'-UTR, consistent with previous studies (Fig. S4A, B) (Zhang and Hamada 2018). GO analysis of genes associated with differential m^6^A modifications showed enrichment in biological processes related to cell proliferation, DNA damage repair, and pathways associated with genome stability (Fig. S4C, D). KEGG analysis revealed similar findings to RNA-seq results, with enrichment in pathways associated with multiple cancer phenotypes, as well as involvement in transcription and translation regulation, DNA replication and damage repair, and cell growth and death (Fig. S4E, F).

**Fig. 3:**
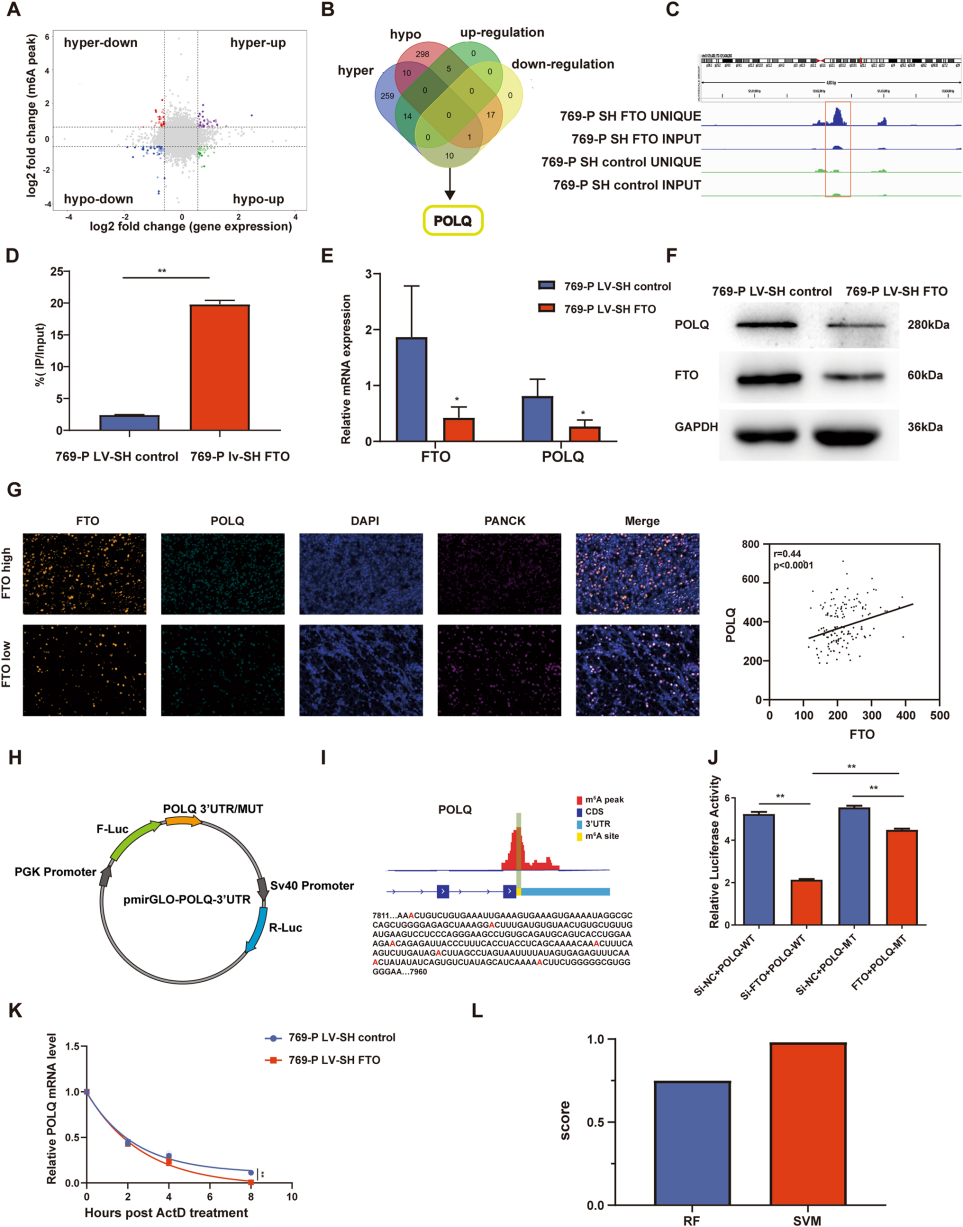
Identification of POLQ as a downstream target of FTO. **A, B** Joint analysis of RNA-seq and MeRIP-seq reveals POLQ as a hyper-downregulated gene. **C** IGV genome visualization software depicts m^6^A methylation levels in the POLQ 3'UTR region in 769-P LV-SH FTO and 769-P LV-SH control cell lines. **D** MeRIP-qPCR detects changes in m^6^A levels on POLQ mRNA following FTO knockdown. **E,F** RT-qPCR and Western blot confirm POLQ expression levels in 769-P LV-SH FTO and 769-P LV-SH control cell lines. **G** Multiplex immunofluorescence staining on ccRCC tissue microarrays demonstrates the correlation between FTO and POLQ expression. **H,I** Prediction of m.^6^A methylation modification sites in the POLQ 3'UTR region using SRAMP and RMBASE databases, followed by the construction of mutant plasmids. **J** Dual-luciferase reporter assays involving pmirGLO-POLQ-3' UTR WT and pmirGLO-POLQ-3' UTR MUT plasmid co-transfected with si-NC and si-FTO. **K** RNA stability analysis of POLQ mRNA abundance in 769-P LV-SH FTO and 769-P LV-SH control cell lines after actinomycin D treatment (8μg/mL). **L** The scores of the interaction probability between POLQ mRNA and YTHDF2 protein predicted by RPIseq. *P < 0. 05, **P < 0. 01

In a comprehensive analysis of RNA-seq and MeRIP-seq results, genes with upregulated peaks were termed "hyper" genes, while genes with downregulated peaks were termed "hypo" genes. Concurrently, genes with upregulated expression were referred to as "up" genes, while genes with downregulated expression were termed "down" genes. A scatter plot was generated to visualize the combined analysis (Fig. 3A). Notably, the hyper-up gene count was 14, hyper-down genes numbered 11, hypo-up genes were 5, and hypo-down genes totaled 18. Cross-referencing differential gene functions in relevant literature and considering that both genes associated with differential peaks and differentially expressed genes were implicated in DDR and DNA replication pathways, we proposed that POLQ might be a crucial target of FTO in ccRCC (Fig. 3B).

Employing the IGV visualization software (hg38 genome assembly), the MeRIP-seq data revealed that upon FTO depletion, a pronounced increase in m^6^A peak intensity was observed within the 3'-UTR region of the POLQ gene (Fig. 3C). MeRIP-qPCR further confirmed that FTO knockdown led to a significant increase in m^6^A modification levels on POLQ mRNA (Fig. 3D).

RNA-seq revealed downregulation of POLQ expression after FTO knockdown, which was confirmed through RT-qPCR (Fig. 3E). Western blot further validated the downregulated POLQ protein expression in 769-P LV-SH FTO (Fig. 3F). Despite observing a reduction in POLQ expression upon FTO knockdown in cell lines, whether this regulatory relationship existed in tissues remained uncertain. Through multiplex immunofluorescence staining on ccRCC tissue microarrays, we observed a significant positive correlation between the expression of FTO and POLQ (Spearman’s *r* = 0.44, *p* < 0.0001) (Fig. 3G).

To substantiate whether FTO impacts POLQ expression through m^6^A, we performed dual-luciferase reporter assays. Based on the knowledge that the differential peak resided near the POLQ 3′ UTR (Fig. 3C), pmirGLO-POLQ-3′ UTR and pmirGLO-POLQ-3′ UTR MUT were constructed (Fig. 3 H,I). Both plasmids harbored the Renilla luciferase gene and the firefly luciferase gene. By using Renilla luciferase as an internal control, the ratio of firefly luciferase luminescence to Renilla luciferase luminescence was calculated to analyze the expression level of POLQ 3′ UTR MUT and WT. Co-transfection of pmirGLO-POLQ-3′ UTR plasmid and pmirGLO-POLQ-3′ UTR MUT plasmid was conducted simultaneously with si-FTO and control si-NC. In the pmirGLO-POLQ-3′ UTR WT group, the ratio of firefly luciferase fluorescence to Renilla luciferase fluorescence significantly decreased after FTO knockdown (Fig. 3J). Although the pmirGLO-POLQ-3′ UTR MUT group also experienced a reduction upon FTO knockdown, the extent of reduction was notably lower compared to the WT group. Thus, it can be reasonably inferred that FTO, to a considerable extent, influences POLQ expression through the regulation of m^6^A methylation.

There is a growing body of evidence suggesting that alterations in m^6^A levels can influence mRNA stability. To investigate whether FTO regulates the expression of POLQ mRNA through this mechanism, we treated 769-P LV-SH FTO and 769-P LV-SH control separately with the transcription inhibitor actinomycin D. RNA was collected at 0, 2, 4, and 8 h post-treatment, and the relative abundance of POLQ mRNA was assessed using RT-qPCR. The results demonstrated that FTO knockdown resulted in decreased stability of POLQ mRNA, leading to a shortened half-life (Fig. 3K).

YTHDF2, as the most prevalent m^6^A reader promoting mRNA degradation, prompted us to predict the interaction between YTHDF2 protein and POLQ mRNA using the RPISeq (http://pridb.gdcb.iastate.edu/RPISeq/) (Muppirala et al. 2011). RPISeq is a family of classifiers designed for predicting RNA–protein interactions based on sequence information, presenting two variants: RPISeq-SVM (Support Vector Machine classifier) and RPISeq-RF (Random Forest classifier). Both classifiers exhibited precision values exceeding 87%, and predictions with probabilities of 0.5 were considered positive. The results indicated an interaction between POLQ mRNA and YTHDF2 protein, with an SVM score of 0.98 and an RF score of 0.75 (Fig. 3L). As YTHDF2 usually facilitates RNA degradation, FTO knockdown leads to an elevation in m^6^A levels on POLQ mRNA. This is highly likely to be recognized by the m^6^A “reader” YTHDF2, causing an accelerated degradation of POLQ mRNA and, consequently, a reduction in POLQ expression."

### FTO knockdown results in increased genomic instability in ccRCC cells and enhances sensitivity to PARPi olaparib

Based on the KEGG and GO analyses conducted using MeRIP-seq and RNA-seq data, the identified pathways consistently showed associations with DNA damage repair. Furthermore, the sequencing data indicated that these pathways were suppressed following FTO knockdown. Notably, FTO knockdown led to a significant decrease in the expression of POLQ, a critical component of the MMEJ pathway. These findings suggest that FTO knockdown may compromise the DNA damage repair mechanisms of 769-P, potentially affecting their ability to maintain genome stability and rendering them more susceptible to DNA damage repair inhibitors. Additionally, previous literature (Ceccaldi et al. 2015; Higgins and Boulton 2018) has reported that concurrent inhibition of POLQ expression during PARPi treatment of tumor cells exhibits a synergistic effect, resulting in the impairment of tumor cells' ability to preserve genomic integrity and subsequent cell death. Given this, we chose to employ the PARPi olaparib in 769-P LV-SH FTO and 769-P LV-SH control to investigate the impact of FTO knockdown on genome stability maintenance in ccRCC cells.

**Fig. 4:**
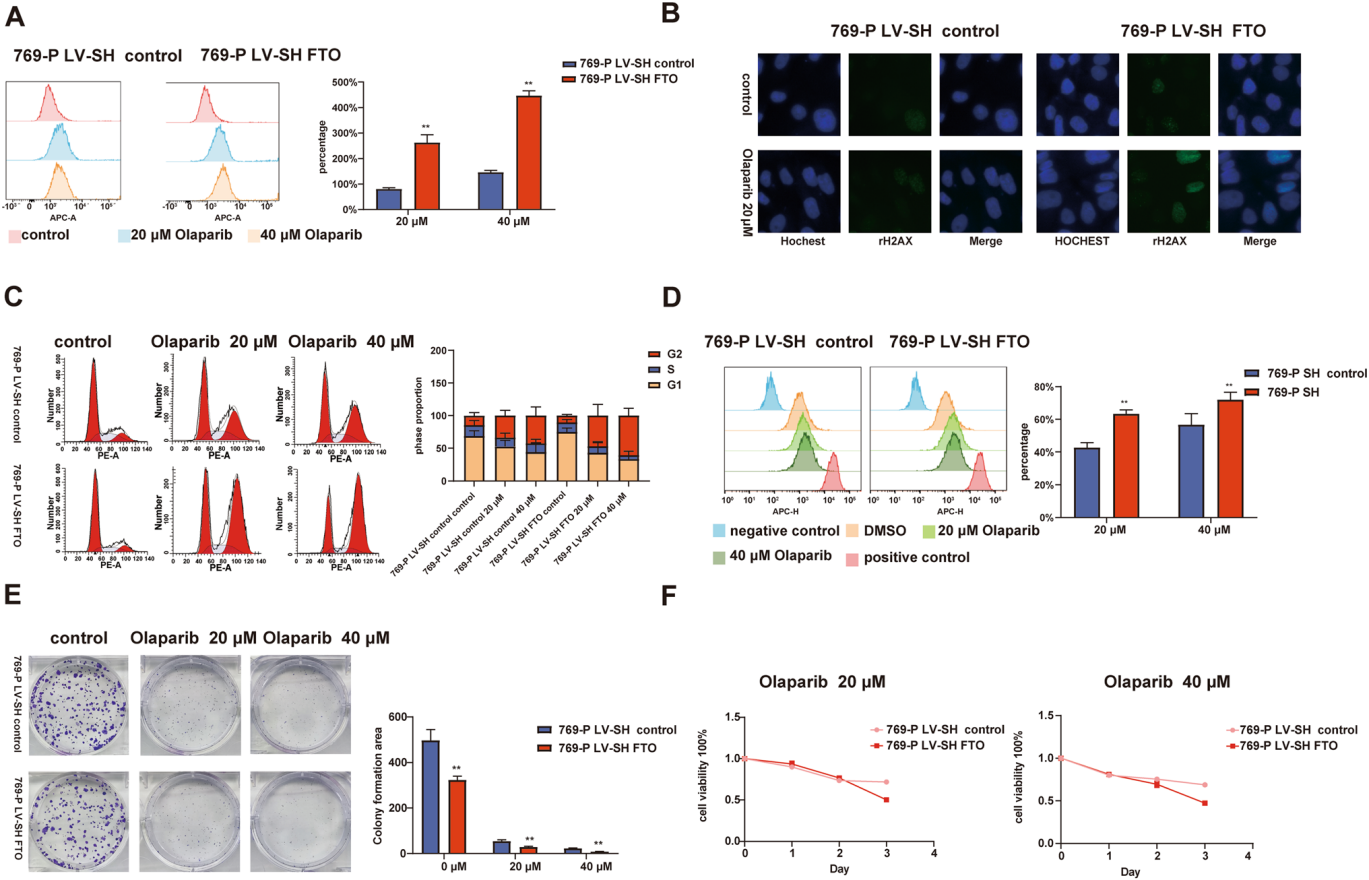
Knockdown of FTO increases DNA damage, cell death, and enhanced proliferation inhibition with olaparib. **A** LIVE/DEAD Fixable Dead Cell Stain flow cytometry assay detecting the proportion of dead cells in 769-P LV-SH FTO and 769-P LV-SH control under olaparib treatment. **B** Immunofluorescence staining of gammaH2AX in 769-P LV-SH FTO and 769-P LV-SH under olaparib treatment. **C** Cell cycle analysis of 769-P LV-SH control and 769-P LV-SH FTO after treatment with 20 μM and 40 μM olaparib. **D** CytoTell 650 flow proliferation assay of 769-P LV-SH control and 769-P LV-SH FTO after treatment with 20 μM and 40 μM olaparib. **E** Colony formation assays of 769-P LV-SH control and 769-P LV-SH FTO after treatment with 20 μM and 40 μM olaparib. **F** CCK-8 cell viability assay of 769-P LV-SH control and 769-P LV-SH FTO after treatment with 20 μM and 40 μM olaparib. *P < 0.05, **P < 0.01

LIVE/DEAD Fixable Dead Cell Stain flow cytometry assay was used to assess cell death caused by olaparib. Under different doses of olaparib, 769-P LV-SH FTO cells exhibited a higher proportion of dead cells (Fig. 4A). Immunofluorescence staining revealed that in the absence of olaparib, there was no significant difference in the expression of the gammaH2AX (DNA double-strand-break marker) between 769-P LV-SH FTO and 769-P LV-SH control. However, after 72 h of olaparib treatment, 769-P LV-SH FTO cells showed a significantly increased expression of gammaH2AX compared to 769-P LV-SH control (Fig. 4B).

Analysis of cell cycle by flow cytometry indicated that FTO knockdown did not induce G2 phase arrest in 769-P. However, it significantly enhanced the G2 phase arrest effect induced by olaparib treatment (Fig. 4C). The impact of 72-h olaparib treatment at 20 μM and 40 μM on cell proliferation was assessed using CytoTell™ Red 650 kit, and consistent with the cell cycle results, 769-P LV-SH FTO exhibited significantly greater inhibition of proliferation (Fig. 4D). Colony formation assays also demonstrated that FTO knockdown enhanced the sensitivity to varying concentrations of olaparib (Fig. 4E). In the CCK-8 cell viability assay, after 72 h of treatment with 20 μM and 40 μM olaparib, the 769-P LV-SH FTO group exhibited notably stronger inhibition (Fig. 4F).

### FTO modulates ccRCC cell proliferation and genome stability via POLQ

**Fig. 5:**
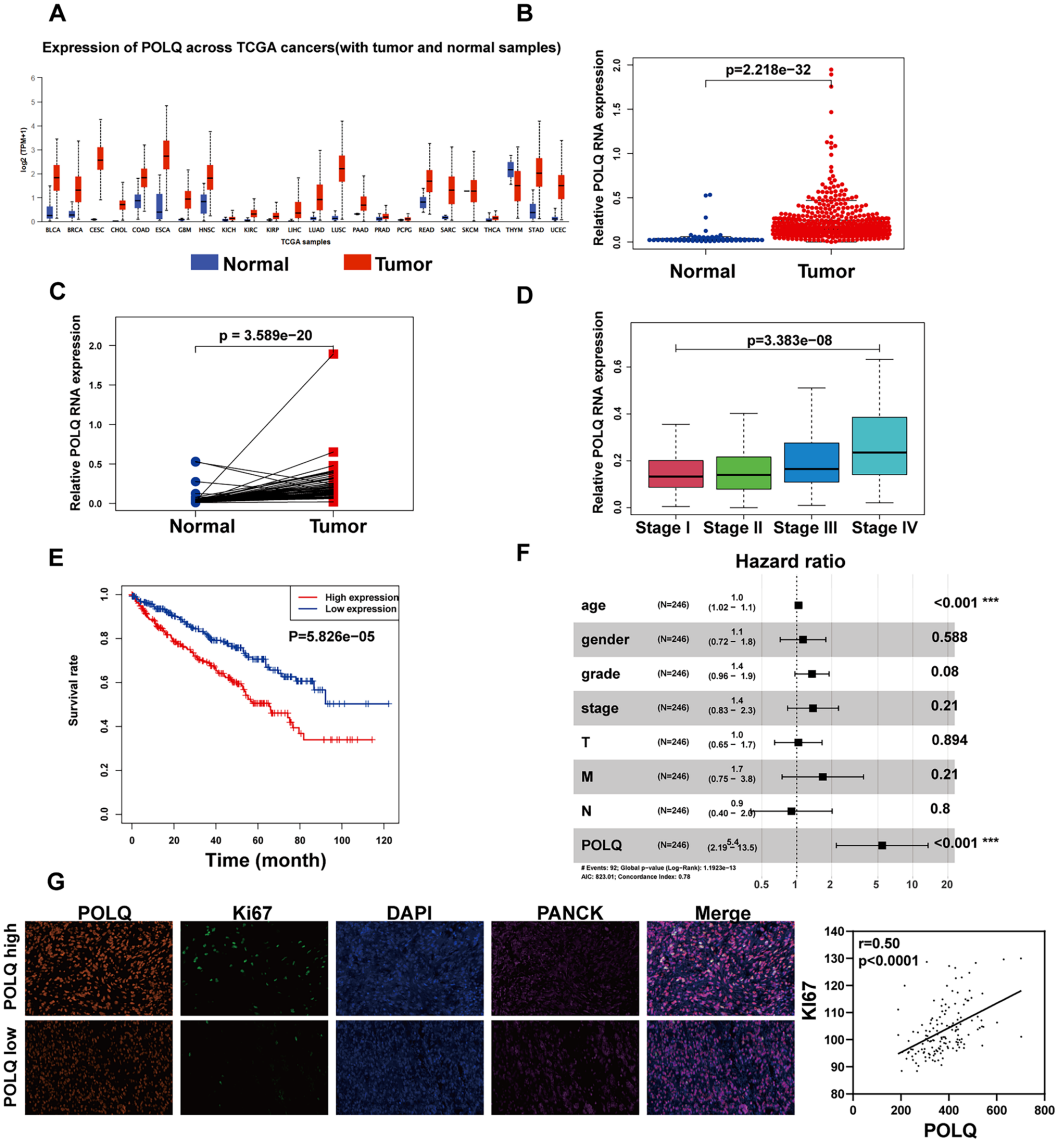
POLQ exhibits increased expression in ccRCC and is associated with the prognosis.** A** Pan-cancer analysis of POLQ. **B, C** Expression of POLQ in ccRCC and adjacent normal tissue. **D** Expression of POLQ in ccRCC tissues at different stages. **E** Survival analysis of POLQ in ccRCC. **F** COX analysis of POLQ in ccRCC.**G** Multiplex immunofluorescence staining on ccRCC tissue microarrays demonstrates the correlation between POLQ and Ki67 expression.*P < 0.05, **P < 0.01

Consistently as anticipated, the pan-cancer analysis from the TCGA public database revealed that POLQ was highly expressed in nearly all tumor types due to increased replication stress and frequent DNA damage within tumors (Fig. 5A). This phenomenon was also observed in ccRCC (Fig. 5B,C). Furthermore, the expression of POLQ in ccRCC increased with tumor stage progression (Fig. 5D). Additionally, survival analysis using the R software "survival" package demonstrated an association between high POLQ expression and adverse prognosis in ccRCC cases (Fig. 5E). Subsequently, a prognostic model was constructed through multifactorial COX analysis for ccRCC, and a forest plot was generated. Notably, POLQ and age emerged as two independent prognostic factors in the multifactorial COX analysis, underscoring the significant impact of POLQ on ccRCC progression and prognosis (Fig. 5F, Table 1). Furthermore, multiplex immunofluorescence staining conducted on ccRCC tissue microarrays also revealed a positive correlation between the expression of POLQ and the proliferation marker Ki67 in ccRCC tissues(Fig. 5G).

**Table 1:** Multivariable cox analysis of POLQ and clinical correlates

	HR	HR.95L	HR.95H	*P* value（** *P*<0.01）
Age	1.04	1.02	1.06	0.0004**
Gender	1.13	0.72	1.78	0.5877
Stage	1.35	0.96	1.9	0.0805
Grade	1.38	0.83	2.29	0.2095
T	1.03	0.65	1.65	0.8937
M	1.68	0.75	3.75	0.2096
N	0.9	0.4	2.02	0.7999
POLQ	5.44	2.19	13.51	0.0003**

**Fig. 6:**
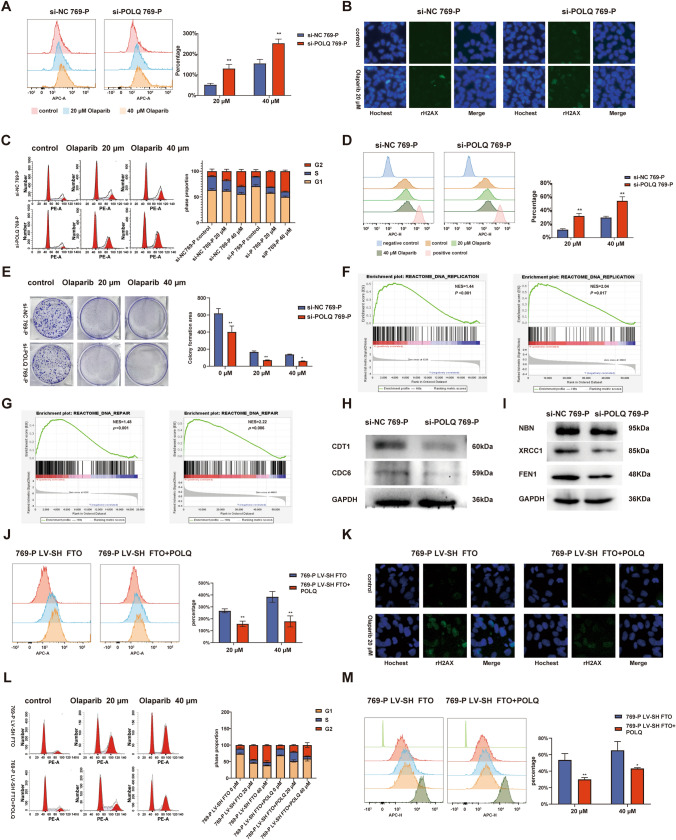
POLQ knockdown results in cell proliferation inhibition and genomic instability in ccRCC.** A** Detection of the proportion of dead cells in si-NC 769-P and si-POLQ 769-P under olaparib treatment using the LIVE/DEAD Fixable Dead Cell Stain flow cytometry assay. **B** Immunofluorescence staining of gammaH2AX in si-NC 769-P and si-POLQ 769-P.** C** Cell cycle analysis of si-NC 769-P and si-POLQ 769-P cells after treatment with 20 μM and 40 μM olaparib. **D** CytoTell 650 flow proliferation assay of si-NC 769-P and si-POLQ 769-P cells after treatment with olaparib. **E** Colony formation assay of si-NC 769-P and si-POLQ 769-P after treatment with olaparib. **F** GSEA enrichment analysis based on RNA-seq data from the LV-SH control and LV-SH FTO groups. **G** GSEA enrichment analysis based on transcriptome TCGA database comparing POLQ high-expression and low-expression groups. **H** CDT1 and CDC6 protein expression level determined by Western blot.** I** NBN, XRCC1, and FEN1 protein expression level determined by Western blot. **J** Detection of the proportion of dead cells in 769-P LV-SH FTO and 769-P LV-SH FTO + POLQ under olaparib treatment using the LIVE/DEAD Fixable Dead Cell Stain flow cytometry assay. **K** Immunofluorescence staining of gammaH2AX in 769-P LV-SH FTO and 769-P LV-SH FTO + POLQ.** L** Cell cycle analysis of 769-P LV-SH FTO and 769-P LV-SH FTO + POLQ after treatment with 20 μM and 40 μM olaparib. **M** CytoTell 650 flow proliferation assay of 769-P LV-SH FTO and 769-P LV-SH FTO + POLQ after treatment with olaparib. *P < 0.05, **P < 0.01

The LIVE/DEAD Fixable Dead Cell Stain assay was employed to observe cellular damage caused by olaparib. Under olaparib treatment, si-POLQ 769-P exhibited a higher proportion of dead cells compared to the si-NC 769-P (Fig. 6A). Immunofluorescence staining of the gammaH2AX showed that in the absence of olaparib treatment, si-POLQ 769-P cells had slightly higher levels of gammaH2AX compared to the si-NC 769-P. However, after 72 h of olaparib treatment, si-POLQ 769-P cells displayed a significantly more pronounced upregulation of gammaH2AX compared to si-NC 769-P (Fig. 6B).

Flow cytometry revealed that in the control group without olaparib treatment, POLQ knockdown resulted in a downregulation of the S-phase population. However, in the olaparib treatment group, POLQ knockdown led to a more severe G2 phase arrest (Fig. 6C). CytoTell 650 flow proliferation assay indicated that si-POLQ 769-P cells exhibited a significantly higher degree of inhibition of cell proliferation compared to si-NC 769-P cells (Fig. 6D). Colony formation assays showed that in the control group, knocking down POLQ resulted in a slower cell proliferation rate, and olaparib treatment further enhanced the inhibition of growth (Fig. 6E).

To explore downstream pathways related to the FTO/POLQ axis, we conducted Gene Set Enrichment Analysis (GSEA) to determine the enriched features of FTO and POLQ in ccRCC. Both GSEA analyses identified common pathways, including DNA replication and DNA damage repair. Specifically, based on RNA-seq results, GSEA analysis revealed enrichment of DNA replication and DNA damage repair pathways in the LV-SH control group (Fig. 6F). Meanwhile, based on TCGA ccRCC transcriptome data, DNA replication and DNA damage repair pathways were enriched in the high POLQ expression group (Fig. 6G). POLQ has also been shown to regulate the expression of DNA replication initiation proteins, including CDC6, CDT1, and CDC45, affecting DNA replication, exacerbating replication stress in tumors, and consequently influencing cell proliferation and prognosis (Allera-Moreau et al. 2012; Goullet de Rugy et al. 2016). Using ccRCC transcriptome data from TCGA database and in conjunction with relevant literature reports, we conducted a comprehensive analysis of the correlation between POLQ and multiple DNA replication initiation proteins, as well as MMEJ pathway molecules. The results revealed that in tumor tissues from ccRCC patients, POLQ expression positively correlated with the expression of CDC6, CDC45, CDT1, FANCD2, and MCM2 (Fig. S5). Additionally, it also exhibited positive correlations with the expression of MMEJ pathway molecules (Fig. S6).Subsequently, we validated these findings through Western blot, the expression of DNA replication initiation proteins CDT1 and CDC6 was downregulated in si-POLQ 769 cells (Fig. 6H). Additionally, several crucial proteins involved in the MMEJ pathway, including NBN, FEN1, and XRCC1, were found to be downregulated in si-POLQ 769-P (Fig. 6I). These results suggest that knocking down POLQ may, in part, regulate cell proliferation in ccRCC by affecting DNA replication initiation and alter the DNA damage repair capacity of ccRCC cells through modulation of the MMEJ pathway, resulting in decreased genome stability.

To further validate the influence of FTO on ccRCC genomic stability and cell proliferation through the modulation of POLQ, we carried out a series of rescue experiments. The assays included LIVE/DEAD Fixable Dead Cell Stain and immunofluorescence staining of gammaH2AX (Fig. 6J, K). The outcomes demonstrated that the overexpression of POLQ reduced the proportion of dead cells and mitigated DNA damage in 769-P LV-SH FTO following olaparib treatment. Flow cytometry indicated that the sole overexpression of POLQ increased the S-phase population in 769-P LV-SH FTO, while G2 phase arrest was alleviated after olaparib treatment (Fig. 6L). In line with the flow cytometry, the CytoTell 650 flow proliferation assay revealed that, in the POLQ overexpression group, olaparib induced a less pronounced proliferation inhibition in 769-P LV-SH FTO (Fig. 6M).

## Discussion

m^6^A is the most widespread epigenetic modification found in eukaryotic RNA, participating in various physiological and pathological processes by influencing RNA processes such as precursor splicing, translation, and degradation. In numerous cancers, there is a consistent observation of dysregulated expression of key enzymes involved in m^6^A modification, including "writers" (such as METTL3, METTL14, WTAP), "erasers" (like FTO, ALKBH5), and "readers" (including YTHDF1, YTHDF2, YTHDF3) (Sun et al. 2019). These enzymes can impact tumor phenotypes by regulating or recognizing the m^6^A methylation modification levels of downstream target genes, exerting multiple effects on cancer progression. Since the discovery of FTO as the first identified m^6^A demethylase enzyme in 2011, it has been demonstrated to play a crucial role in influencing cancer progression by removing m^6^A modifications from downstream target genes in various cancers (Huang et al. 2022; Niu et al. 2019). However, FTO's role in these cancers varies between a tumor suppressor or an oncogene. This diversity in roles is attributed, in part, to the differences in major downstream target genes of FTO and the distinct actions of m^6^A “readers” on downstream target genes. FTO has been confirmed to affect biological processes in ccRCC, including the regulation of mitochondrial function, cellular autophagy, and glutamine transport (Shen et al. 2022; Xiao et al. 2020; Xu et al. 2022; Zhang et al. 2021; Zhuang et al. 2019). However, its specific role as a promoter or inhibitor of cancer remains a topic of debate. This may necessitate further validation using a more unified tumor model. To our knowledge, the mechanisms underlying FTO's regulation of genome stability in ccRCC have not been reported. Recently, there has been a notable shift in focus toward DDR-related genes as promising targets for ccRCC therapy. Mutations in DDR-related genes have been identified as factors that can heighten the sensitivity of ccRCC to targeted agents such as sunitinib (Hsieh et al. 2017). Additionally, the inhibition of DDR-related genes has been associated with prolonged survival in ccRCC patients undergoing immune ICI therapy (Hagiwara et al. 2022). Taking into consideration the dependency of tumor cells on DDR, the utilization of DDR inhibitors in cancer treatment not only serves to suppress tumor cells but also minimizes damage to normal cells. In this context, the present study illuminates the regulatory role of FTO in influencing the DDR function of ccRCC cells by modulating the expression of POLQ. These findings hold potential implications for the advancement of precise therapies and the development of innovative combination strategies for ccRCC patients.

In this study, we have observed elevated expression of FTO in ccRCC cell lines and tumor tissues. Notably, high FTO expression in tumor tissues holds diagnostic significance and has the potential to become one of the diagnostic biomarkers for ccRCC. FTO exerted a pronounced inhibitory effect on ccRCC cell proliferation, which was substantiated through CCK-8 assays, colony formation assays, and cell cycle analysis. Additionally, as a crucial enzyme involved in the removal of m^6^A methylation modifications, FTO knockdown led to an overall increase in m^6^A levels in ccRCC cell lines. Through overexpression of FTO^MUT^ and FTO^WT^, it was revealed that FTO primarily influences ccRCC cell proliferation by modulating m^6^A modifications. MeRIP-seq and RNA-seq sequencing were employed to identify downstream target genes of FTO in ccRCC. Sequencing results identified that FTO regulates the expression of POLQ by modulating m^6^A modifications, with m^6^A modification sites located in the 3'UTR region. This conclusion was further validated through dual-luciferase reporter assays, MeRIP-qPCR, RT-qPCR, and Western blot.

POLQ is a pivotal protein in the MMEJ pathway, which, despite initially being considered as a backup pathway for DSB repair, has been demonstrated to be indispensable for cell survival in certain contexts (Goullet de Rugy et al. 2016). Elevated expression of POLQ has been observed in various tumor tissues, such as lung cancer, colon cancer, and gastric cancer, where it contributes to maintaining genome stability and correlates with poor patient prognosis (Kawamura et al. 2004). In HR-deficient tumors, POLQ also exhibits higher expression levels, highlighting its essential role in DDR (Ceccaldi et al. 2015). Previous literature reports have indicated that POLQ promotes cell growth and malignant progression in various cancers, including liver cancer, cervical cancer, breast cancer, and colon cancer (Allera-Moreau et al. 2012; Lemee et al. 2010; Pan et al. 2021; Pillaire et al. 2010; Shinmura et al. 2019). In non-small cell lung cancer, POLQ upregulates DNA replication initiation proteins such as CDC6 and PLK1, enhancing tumor cell tolerance to replication stress, promoting cell proliferation, and resulting in poor prognosis (Allera-Moreau et al. 2012). In breast cancer and colon cancer, POLQ is associated with the upregulation of genome stability genes and replication initiation proteins such as CDC6, CDT1, and CDC45 (Goullet de Rugy et al. 2016). These studies suggest that overexpression of POLQ in cancer not only maintains genome stability but also participates in the regulation of DNA replication initiation, influencing tumor cell proliferation.

In this study, TCGA pan-cancer analysis revealed elevated expression of POLQ in various cancers, including ccRCC, and its expression levels were correlated with tumor stage. High POLQ expression was associated with poor patient prognosis and served as an independent prognostic factor in ccRCC. POLQ knockdown resulted in reduced DDR capacity, compromised genome stability, and inhibited cell proliferation in ccRCC. Additionally, GSEA analysis based on RNA-seq and TCGA data showed enrichment of DNA replication and DNA damage repair pathways in both FTO and POLQ high-expression groups. Western blot revealed downregulation of replication initiation proteins CDC6 and CDT1 after POLQ knockdown. CDC6 has been confirmed to promote ccRCC cell proliferation and influence cell cycle regulation (Chen et al. 2022; Liu et al. 2020). Furthermore, Western blot showed downregulation of MMEJ pathway proteins XRCC1, NBN, and FEN1 after POLQ knockdown, confirming inhibition of the DDR pathway MMEJ.

Olaparib is a PARPi that was initially approved for the treatment of breast and cervical cancers with BRCA1 or BRCA2 functional deficiencies, inducing a synthetic lethality effect. Recent research has indicated that PARPi may also be effective in tumors with "BRCAness," where HR functions are impaired, even in the absence of BRCA1 or BRCA2 mutations. Approximately one-third of ccRCC samples were found to have mutations associated with HR genes (Pletcher et al. 2021), supporting the potential utility of olaparib as a therapeutic option for ccRCC.

**Fig. 7:**
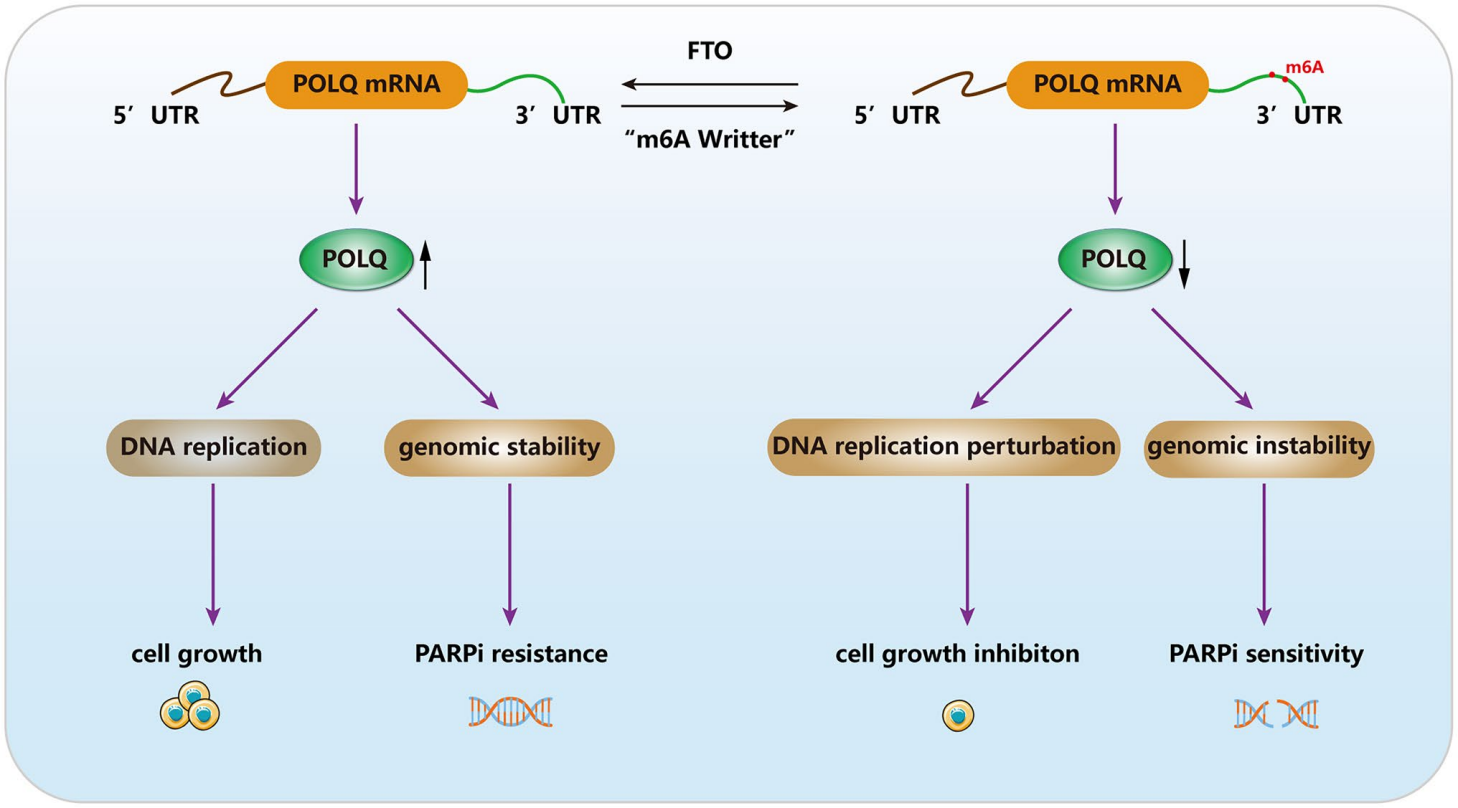
FTO promotes ccRCC cell proliferation and genome stability by regulating POLQ

Furthermore, previous studies have explored the potential mechanisms underlying PARPi's inhibition of RCC progression and investigated potential combination therapies with other drugs (Lai et al. 2022). A significant discovery is that inhibiting POLQ in combination with PARPi can more effectively impair the survival capacity of tumor cells and compromise their DNA damage repair capability (Lavudi et al. 2023). In our study, knocking down either FTO or POLQ also increased the sensitivity of ccRCC cells to olaparib, although the precise regulatory mechanisms require further elucidation. The primary outcomes of this study have been summarized in the schematic representation in Fig. 7.

## Conclusion

Here, we report that FTO, through the regulation of m^6^A modifications, influences the expression of POLQ, thereby promoting the cell proliferation and DNA damage repair capacity of ccRCC cells while maintaining genome stability. This discovery unveils a novel molecular mechanism of FTO in the progression of ccRCC, holding promise for personalized treatments in ccRCC patients. Moreover, it presents a potential novel treatment strategy for those ccRCC patients who have developed resistance to first-line treatment.

## Supplementary Information

Below is the link to the electronic supplementary material.

Supplementary file1 (DOCX 5798 KB)

## Data Availability

All data used in this study are included in the article. Please contact the corresponding author for data requests.
