# Supplementary material for: The m6A demethylase FTO targets POLQ to promote ccRCC cell proliferation and genome stability maintenance
Source: J Cancer Res Clin Oncol. 2024 Jan 25;150(2):30. doi: 10.1007/s00432-023-05541-0 (PMC10810938; doi:10.1007/s00432-023-05541-0)
Supplement: Supplementary file 1 — Supplementary file1 (DOCX 5798 KB) [file 432_2023_5541_MOESM1_ESM.docx]

# m**^6^**A demethylase FTO promotes ccRCC cell proliferation and genome stability by regulating POLQ

Yichen He^1,2,3^,Yimeng Chen^3^,Zhengsheng Li ^3^,Changping Wu^1,2*^

**Affiliations**

^1^Department of Tumor Biological Treatment, The Third Affiliated Hospital of Soochow University, Changzhou 213003, China. Email: 20194035003@stu.suda.edu.cn (Yichen He); [wcpjjt@163.com](mailto:wcpjjt@163.com) (Changping Wu)

^2^Institute of Cell Therapy, The Third Affiliated Hospital of Soochow University, Changzhou 213003, China. Email: 20194035003@stu.suda.edu.cn (Yichen He); [wcpjjt@163.com](mailto:wcpjjt@163.com) (Changping Wu)

^3^Department of Urology, The Third Affiliated Hospital of Soochow University, Changzhou 213003, China. Email: 20194035003@stu.suda.edu.cn (Yichen He); [wcpjjt@163.com](mailto:wcpjjt@163.com) (Changping [Wu);chenyimeng@sibs.ac.cn](mailto:Wu);chenyimeng@sibs.ac.cn) (Yimeng Chen); 326944672@qq.com (Zhengsheng Li)

***Correspondence**

Changping Wu

[wcpjjt@163.com](mailto:wcpjjt@163.com)


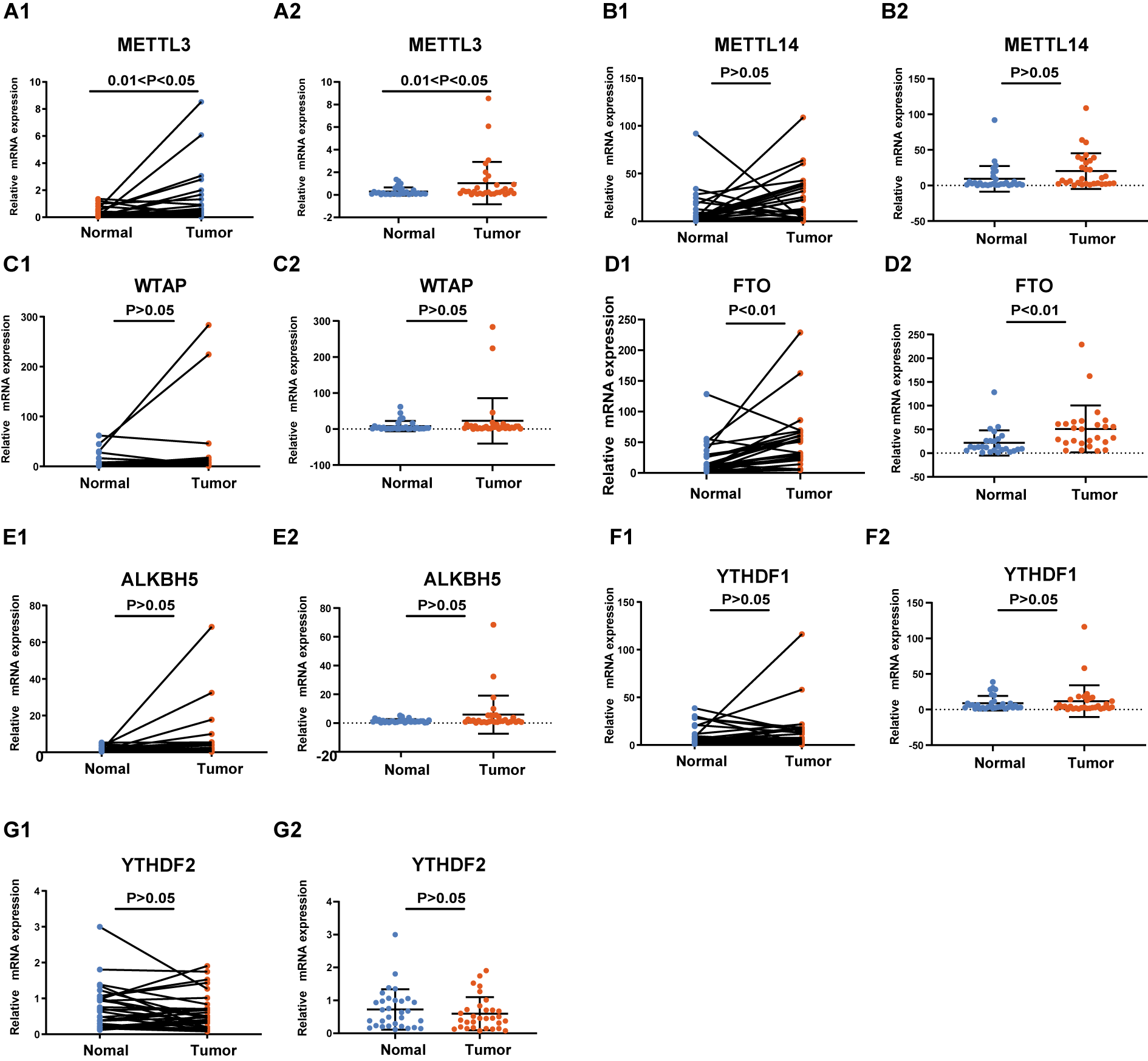


**Figure. S1** The mRNA expression levels of key enzymes involved in m^6^A modification in ccRCC clinical samples determined by RT-qPCR. **A1, A2** Expression levels of METTL3 mRNA in cancer tissue and adjacent normal tissue. **B1, B2** Expression levels of METTL14 mRNA in cancer tissue and adjacent normal tissue.

**C1, C2** Expression levels of WTAP mRNA in cancer tissue and adjacent normal tissue. **D1, D2** Expression levels of FTO mRNA in cancer tissue and adjacent normal tissue. **E1, E2** Expression levels of ALKBH5 mRNA in cancer tissue and adjacent normal tissue. **F1, F2** Expression levels of YTHDF1 mRNA in cancer tissue and adjacent normal tissue. **G1, G2** Expression levels of YTHDF2 mRNA in cancer tissue and adjacent normal tissue.


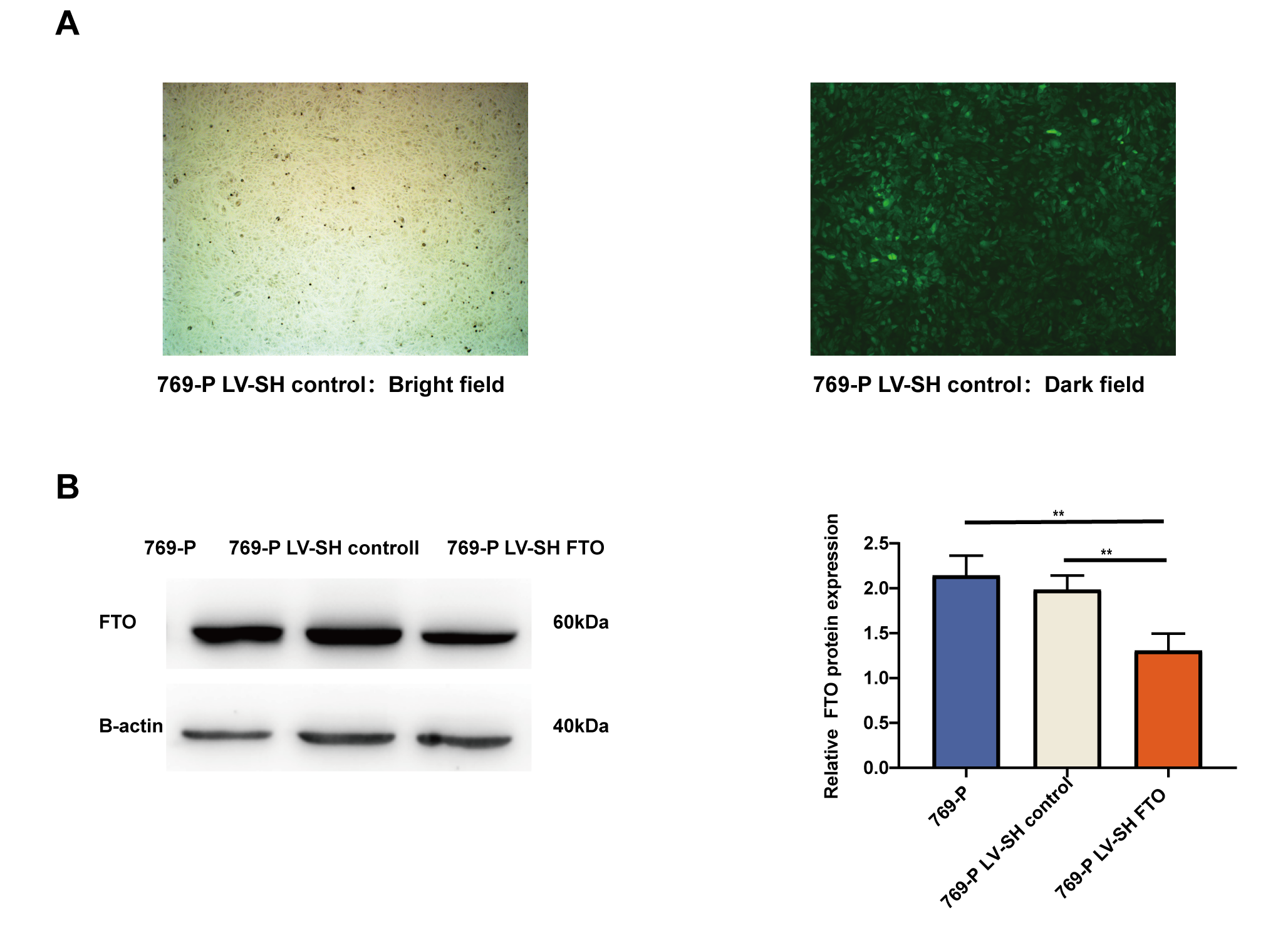


**Figure. S2** Infection efficiency and knockdown efficiency validatio. **A** The same vision field was observed under white light and fluorescence. **B** The efficiency of knockdown was determined by Western blot.


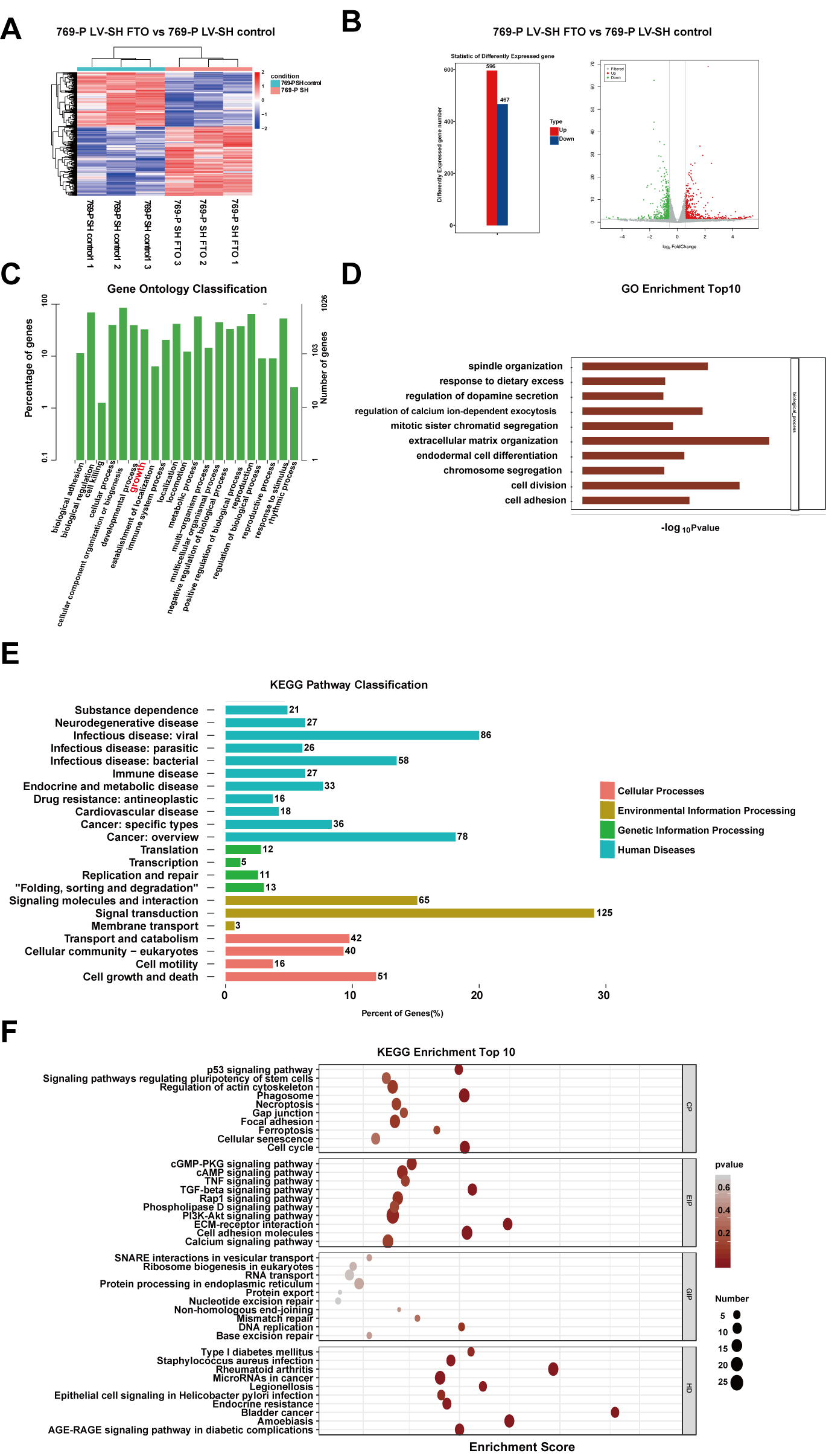


**Figure. S3** Analysis of differentially expressed genes. **A** Clustering analysis of differentially expressed genes. **B** Count of differentially expressed genes. **C, D** Gene Ontology (GO) analysis of differentially expressed genes. **E, F** Kyoto Encyclopedia of Genes and Genomes (KEGG) analysis of differentially expressed genes.


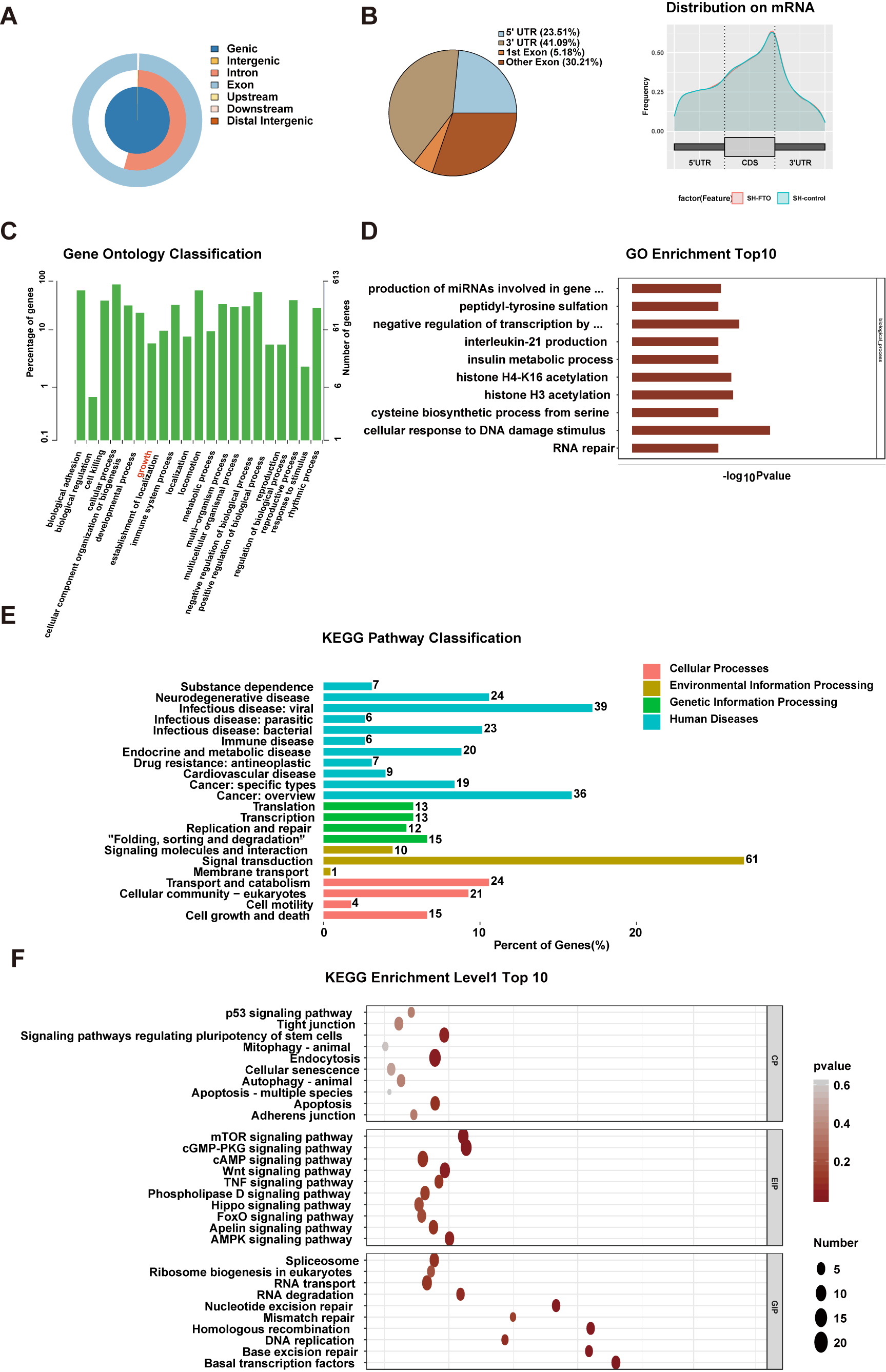


**Figure. S4** Analysis of differential m^6^A peak-related genes. **A,B** Analysis of the locations of differential m^6^A peaks. **C, D** Gene Ontology (GO) analysis of differential m^6^A peak-related genes. **E, F** Kyoto Encyclopedia of Genes and Genomes (KEGG) analysis of differential m^6^A peak-related genes.


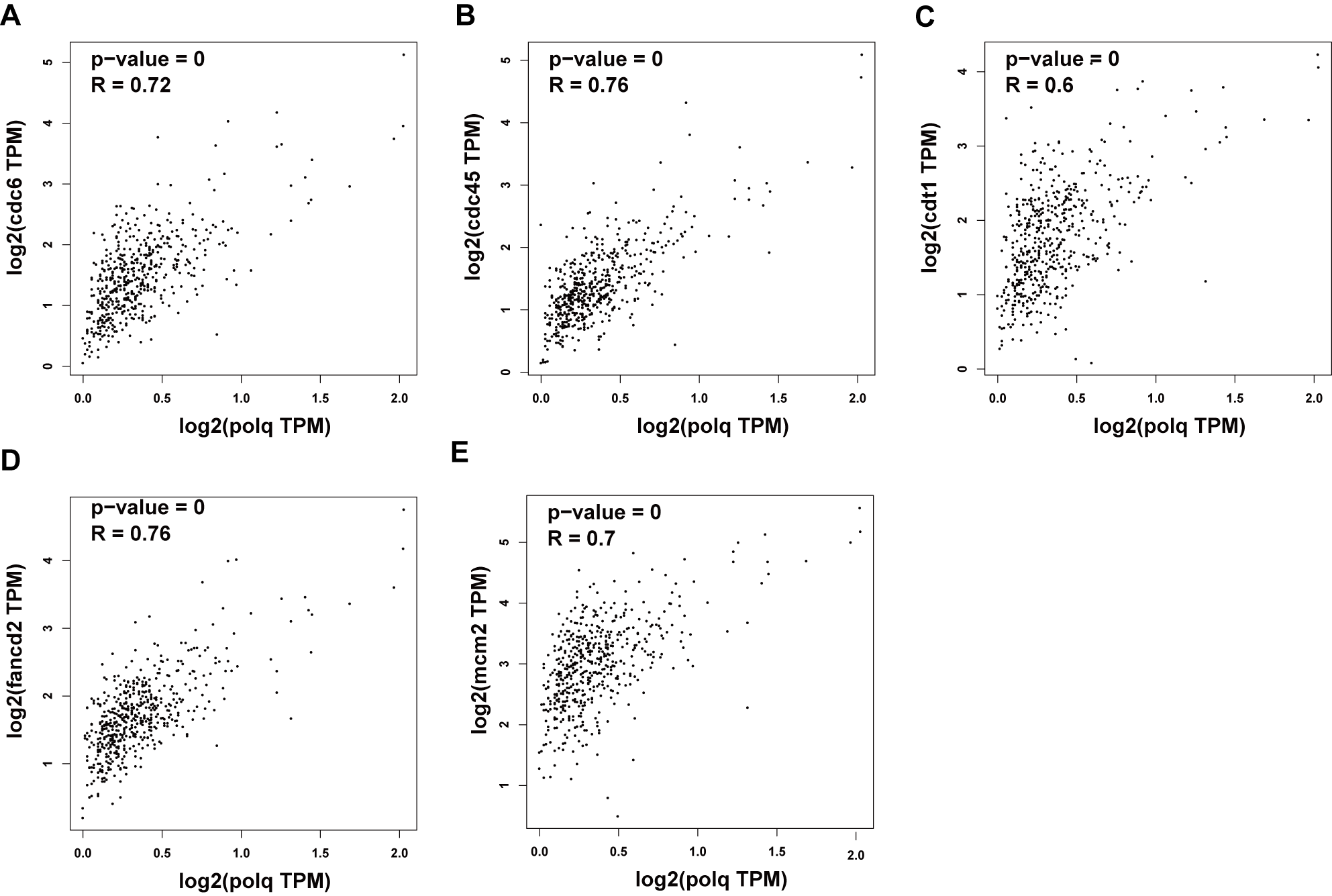


**Figure. S5** The expression correlation of POLQ with CDC6 (A), CDC45 (B), CDT1 (C), Fancd2 (D), and MCM2 (E).


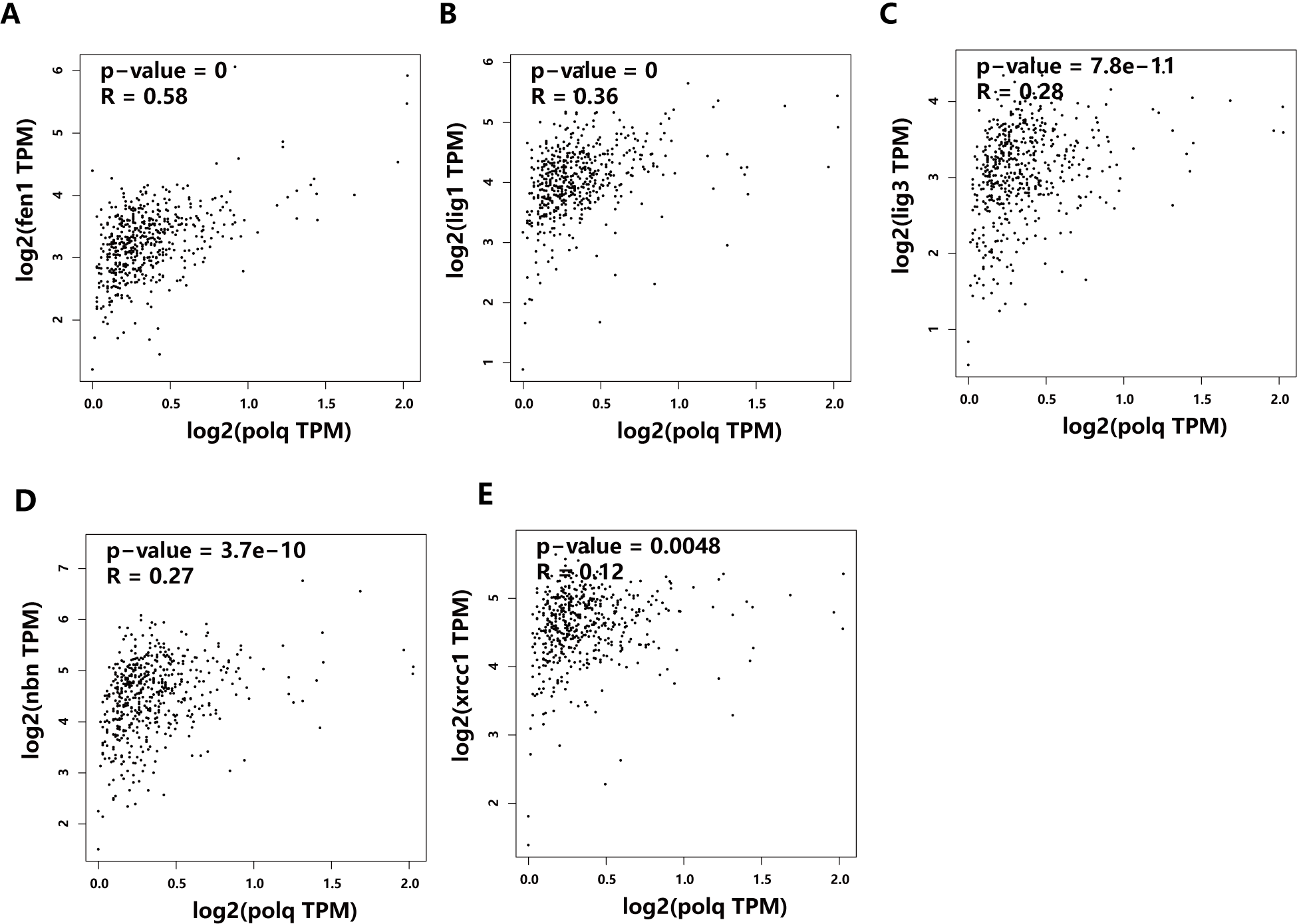


**Figure. S6** The expression correlation of POLQ with FEN1 (A), LIG1 (B), LIG3 (C), NBN (D), and XRCC1 (E).
